# Size Distribution and Pathogenic Potential of Culturable Airborne *Clostridium* spp. in a Suburb of Toyama City, Japan

**DOI:** 10.1264/jsme2.ME24078

**Published:** 2025-02-06

**Authors:** Makoto Seki, Reika Iwamoto, Jianjian Hou, So Fujiyoshi, Fumito Maruyama, Yukihiro Furusawa, Shigehiro Kagaya, Akihiro Sakatoku, Shogo Nakamura, Daisuke Tanaka

**Affiliations:** 1 Graduate School of Science and Engineering, University of Toyama, 3190 Gofuku, Toyama, Toyama 930–8555, Japan; 2 Center for the Planetary Health and Innovation Science (PHIS), The IDEC Institute, Hiroshima University, 1–3–2 Kagamiyama, Higashi-Hiroshima, Hiroshima 739–8511, Japan; 3 Department of Pharmaceutical Engineering, Faculty of Engineering, Toyama Prefectural University, 5180 Kurokawa, Imizu, Toyama 939–0398, Japan

**Keywords:** bioaerosols, *Clostridium*, sporobiota, virulence genes, antibiotic resistance genes

## Abstract

*Clostridium* spp. are anaerobic, Gram-positive, spore-forming bacteria comprising more than 150 species, some of which are important pathogens of humans and animals. Members of this genus have been isolated from a number of environments, but are rarely found in the atmosphere. In the present study, we exami­ned culturable airborne *Clostridium* spp. and clarified their pathogenicity. We obtained 19 culturable *Clostridium* isolates from size-fractionated samples collected at a suburban site in Toyama, central Japan. Culturable *Clostridium* spp. were detected in particles larger than 1.1‍ ‍μm, and the size distribution peaked at 2.1–3.3‍ ‍μm, corresponding to the spore size of *Clostridium* spp. More *Clostridium* spp. were detected in coarse particles >2.1‍ ‍μm not only by culture methods, but also by 16S rRNA gene amplicon sequencing. Whole-genome sequencing (WGS) identified seven *Clostridium* species, among which *Clostridium perfringens* was predominant. Moreover, WGS revealed that *C. perfringens* isolates harbored many virulence and antibiotic resistance genes with the potential to cause gas gangrene. The detection and characterization of potential airborne pathogens are crucial for preventing the spread of diseases caused by these pathogens. To the best of our knowledge, this is the first study to demonstrate that anaerobic *Clostridium* spp. may be transported under aerobic conditions in the atmosphere and pose potential risks to human health.

Particle matter originating from biological substances (known as bioaerosols) is a mixture of dust, microbes, and their fragments (Humbal *et al.*, 2018; Gollakota *et al.*, 2021). Particle sizes range from 0.001–100‍ ‍μm (Kim *et al.*, 2018). Exposure to bioaerosols may have adverse health effects on humans and animals, such as allergies and infections typically involving the respiratory system (Humbal *et al.*, 2018; Maki *et al.*, 2022). Microbial size distribution is important in studies on health risk assessments of exposure to airborne microbes because particle sizes define the inhalable degree of particle matter, which has a crucial effect on human health (Zhai *et al.*, 2018). A relationship has been observed between particulate matter air pollution and gastrointestinal diseases because particulate matter may be indirectly deposited in the oropharynx via mucociliary clearance and swallowed with saliva and mucus (Mutlu *et al.*, 2018; Van Pee *et al.*, 2023). Size-fractionated sampling was shown to efficiently identify the composition of airborne microorganisms, including pathogens (Tanaka *et al.*, 2020; Ferguson *et al.*, 2021; Stern *et al.*, 2021). We previously detected that *Legionella* spp., the causative bacteria of human Legionnaires’ disease, mainly in coarse particles >2.1‍ ‍μm (Tanaka *et al.*, 2020).

*Clostridium* spp. are diverse and include a large group of anaerobic (or aerotolerant), Gram-positive, spore-forming bacteria from more than 150 species, some of which are important pathogens of humans and other animals (Kieu *et‍ ‍al.*, 2021; Li *et al.*, 2023). Four main pathogenic *Clostridium* species cause fatal illnesses: *Clostridium tetani* causes tetanus; *Clostridium botulinum* causes botulism; *C. perfringens* causes gas gangrene and food poisoning; and *Clostridioides difficile* (formerly known as *Clostridium difficile*) causes pseudomembranous colitis (Brüggemann, 2005; Schaumann *et al.*, 2018; Shen *et al.*, 2019). These species have been isolated from diverse environments, including soil, feces, decaying vegetation, the intestinal tracts of humans and animals, marine and freshwater sediments, and water (Shin *et al.*, 2018; Shen *et al.*, 2019; Liu *et al.*, 2020; Seki *et al.*, 2022). *Clostridium* spp. have been detected in the atmosphere by culture-independent mole­cular approaches and were found to be more abundant in coarse particles (2.5–10‍ ‍μm) than in fine particles (<2.5‍ ‍μm) (Leski *et al.*, 2011; Krishnamurthi and Chakrabarti, 2013; Liu *et al.*, 2018; Ruiz-Gil *et al.*, 2020; Tanaka *et al.*, 2020; Ferguson *et al.*, 2021). *Clostridium* spp. have also been identified in the atmosphere using culture methods (Noble *et al.*, 1963; Keessen *et al.*, 2011). However, to the best of our knowledge, a genome ana­lysis of these isolates has not yet been performed to investigate their potential impact on human health.

The aim of the present study was to examine the potential health risks of airborne *Clostridium* spp. in the atmosphere. To achieve this, we isolated culturable airborne *Clostridium* spp. and assessed their size distribution, diversity, and the presence of virulence genes and antibiotic resistance genes (ARGs). We conducted 39 air sampling campaigns using size-fractionated samplers in a suburban site of Toyama in central Japan over a 2-year period. Food poisoning caused by *C. perfringens* has occasionally occurred in Toyama (Nakamura *et al.*, 2004; Tanaka *et al.*, 2007). Culturable *Clostridium* spp. were isolated from the samples collected and whole-genome sequencing (WGS) was performed to evaluate phylogenetic positions and identify virulence genes and ARGs. Bacterial communities in air samples were also analyzed by amplicon sequencing to investigate the relative abundance of *Clostridium* spp. The results obtained provide novel insights into anaerobic spore-forming bacteria (sporobiota) (Xu *et al.*, 2023) in atmospheric environments.

## Materials and Methods

### Sample collection

Thirty-nine air sampling campaigns were conducted between April 2021 and March 2023, one to three times each month, in a suburban site in Toyama city, the capital of Toyama Prefecture, Japan (Table S1) (Tanaka *et al.*, 2015, 2019, 2020). The study site was located on the roof of a three-story building at the Faculty of Science, University of Toyama, in a western suburb of the city (36° 41′ 54″ N, 137° 11′ 13″ E; 23‍ ‍m above mean sea level). An eight-stage Andersen cascade impactor (AN-200; Sibata Scientific) was used to collect air samples, splitting them into eight size fractions based on their aerodynamic diameter (d_a_) as follows: stage 0, >11.0‍ ‍μm; stage 1, 7.0–11.0‍ ‍μm; stage 2, 4.7–7.0‍ ‍μm; stage 3, 3.3–4.7‍ ‍μm; stage 4, 2.1–3.3‍ ‍μm; stage 5, 1.1–2.1‍ ‍μm; stage 6, 0.65–1.1‍ ‍μm; and stage 7, 0.43–0.65‍ ‍μm. The impactor was operated at a flow rate of 28.3 L‍ ‍min^–1^ over a 23-h period (11:00 to 10:00 the next day). Samples were collected on sterilized quartz fiber filters of 80‍ ‍mm in diameter (2500QAT-UP; Tokyo Dylec). To avoid contamination, the sample filter holder and materials used for filter replacement were treated with 70% ethanol or dry-heat sterilization before being used.

### Isolation of *Clostridium* spp.

Half of the filter sample collected was inoculated into deaerated cooked meat medium (Becton, Dickinson and Company) and incubated at 37°C. After 1–2‍ ‍d, cultures with turbidity and gas production were spread on modified GAM agar (Nissui Pharmaceutical) and incubated anaerobically using the AnaeroPack (Mitsubishi Gas Chemical) at 37°C for 1–2 d. Cooked meat medium and modified GAM agar are antibiotic-free and culture a wide variety of anaerobic bacteria. Isolated colonies were selected, transferred into deaerated cooked meat medium, and incubated anaerobically at 37°C for 1–2 d. Cultures exhibiting turbidity and gas production were analyzed for the presence of *Clostridium* spp. using 16S rRNA gene sequencing and genome sequencing.

### 16S rRNA gene sequencing and phylogenetic ana­lysis

Genomic DNA was extracted from bacterial cells through immersion in boiling water for 10‍ ‍min. 16S rRNA genes were amplified by PCR with the bacterial universal primers 27f and‍ ‍1492r (Lane, 1991). PCR products were purified using a QIAquick PCR Purification Kit (Qiagen) and sequenced directly with a BigDye Terminator v3.1 Cycle Sequencing Kit (Applied Biosystems, Foster city) on a 3500 Genetic Analyzer (Applied Biosystems). The sequences obtained were subjected to a search using the Basic Local Alignment Search Tool (BLAST) program on the NCBI website (https://blast.ncbi.nlm.nih.gov). A phylogenetic tree was constructed using MEGA v11 (Tamura *et al.*, 2021) with the neighbor-joining method (Saitou and Nei, 1987) using Kimura 2-parameter distances. The tree was displayed using iTOL (Letunic and Bork, 2021). The reliability of the tree topology was assessed by 1,000 bootstrap resampling.

### WGS and ana­lyses

Genomic DNA was extracted from pure bacterial cultures using a DNeasy Blood & Tissue Kit (Qiagen). The total DNA concentration was measured using a Quant-iT PicoGreen dsDNA Assay Kit (Thermo Fisher Scientific) with a DS-11 FX+ spectrophotometer/fluorometer (DeNovix). A sequencing library was prepared from 1‍ ‍ng of RNA-free genomic DNA using an Illumina DNA Prep kit (Illumina). The library quality was evaluated using an Agilent 4150 Tape-Station System (Agilent) with the Agilent D1000 ScreenTape assay. The library was sequenced using a MiniSeq instrument (Illumina) and MiniSeq High Output Reagent Cartridge (300 cycles) with outputs of 150-bp paired-end reads. Genome data were assembled using A5-miseq pipeline v20160825 (Coil *et al.*, 2015). The automated multi-locus species tree (autoMLST; https://automlst.ziemertlab.com/) (Alanjary *et al.*, 2019) was used to generate a phylogeny in the “*de novo* mode” with the default settings. The multi-locus sequence alignments generated using autoMLST were imported into MEGA v11 and a maximum-likelihood tree was constructed using 32 marker genes with 1,000 bootstrap replicates. Average nucleotide identity (ANI) values were calculated using the ANI calculator with default parameters (http://enve-omics.ce.gatech.edu/ani/) (Gosselin *et al.*, 2022). Digital DNA–DNA hybridization (dDDH) values (Goris *et al.*, 2007) were computed using formula 2 of the Genome-to-Genome Distance Calculator (GGDC) v3.0 (https://ggdc.dsmz.de/home.php) (Auch *et al.*, 2010). Genome sequence data were uploaded to the Type (Strain) Genome Server (TYGS) (https://tygs.dsmz.de/) for genome-based classification and identification. Virulence factor genes were predicted with the Virulence Factor Database (VFDB) (http://www.mgc.ac.cn/VFs/) (Liu *et al.*, 2022), and ARGs were predicted using the Resistant Gene Identifier tool of the Comprehensive Antibiotic Resistant Database (CARD) v3.2.8 (https://card.mcmaster.ca/) (Alcock *et al.*, 2023) and ResFinder v4.4.1 (https://cge.food.dtu.dk/services/ResFinder/) (Zankari *et al.*, 2012). WGS data have been deposited in the DNA Data Bank of Japan (DDBJ) database under accession number NSUB001450.

### 16S rRNA gene amplicon sequencing

Genomic DNA was extracted from one-eighth of the 80-mm-wide filter samples using the FastDNA SPIN kit for Soil (MP Biomedicals). Libraries for high-throughput sequencing of the V3–V4 region of the bacterial 16S rRNA gene were prepared using the primers 341F and 805R, which are described in the Illumina standard protocol (15044223 Rev. B) (Tanaka *et al.*, 2020; Yamanouchi *et al.*, 2022). First PCR (35 cycles) and second PCR (10 cycles) products were purified by Agencourt AMPure XP (Beckman Coulter). Negative controls for the PCR assays contained water instead of template DNA. To prevent potential contamination, these processes were performed in a laminar airflow clean bench. The DNA concentration of the prepared sequencing libraries was quantified using a Qubit 3.0 Fluorometer (Thermo Fisher Scientific) and GenNext NGS Library Quantification Kit (Toyobo). The libraries were pooled in equimolar amounts and sequenced using the 2×250-bp paired format on a MiSeq platform (Illumina) with MiSeq Reagent Nano Kit v2 in the 500-cycles mode. The sequence data obtained were processed using DADA2 v1.28 and analyzed with the Quantitative Insights into Microbial Ecology 2 (QIIME2) v2023.5.0 software package (Fung *et al.*, 2021). Amplicon sequence variants (ASVs) were generated using DADA2 and taxonomically classified using the SILVA 138_99 database. In the 16S rRNA gene fragment ana­lysis, ASVs classified as chloroplasts or mitochondria were removed. Statistical ana­lyses were conducted using the vegan package (http://vegan.r-forge.r-project.org/) in R v4.2.3 (www.r-project.org). 16S rRNA gene amplicon sequencing data have been deposited in the DDBJ database under accession number DRA017675.

## Results

### Isolation of airborne *Clostridium* and 16S rRNA gene phylogeny

**Fig. 1.**
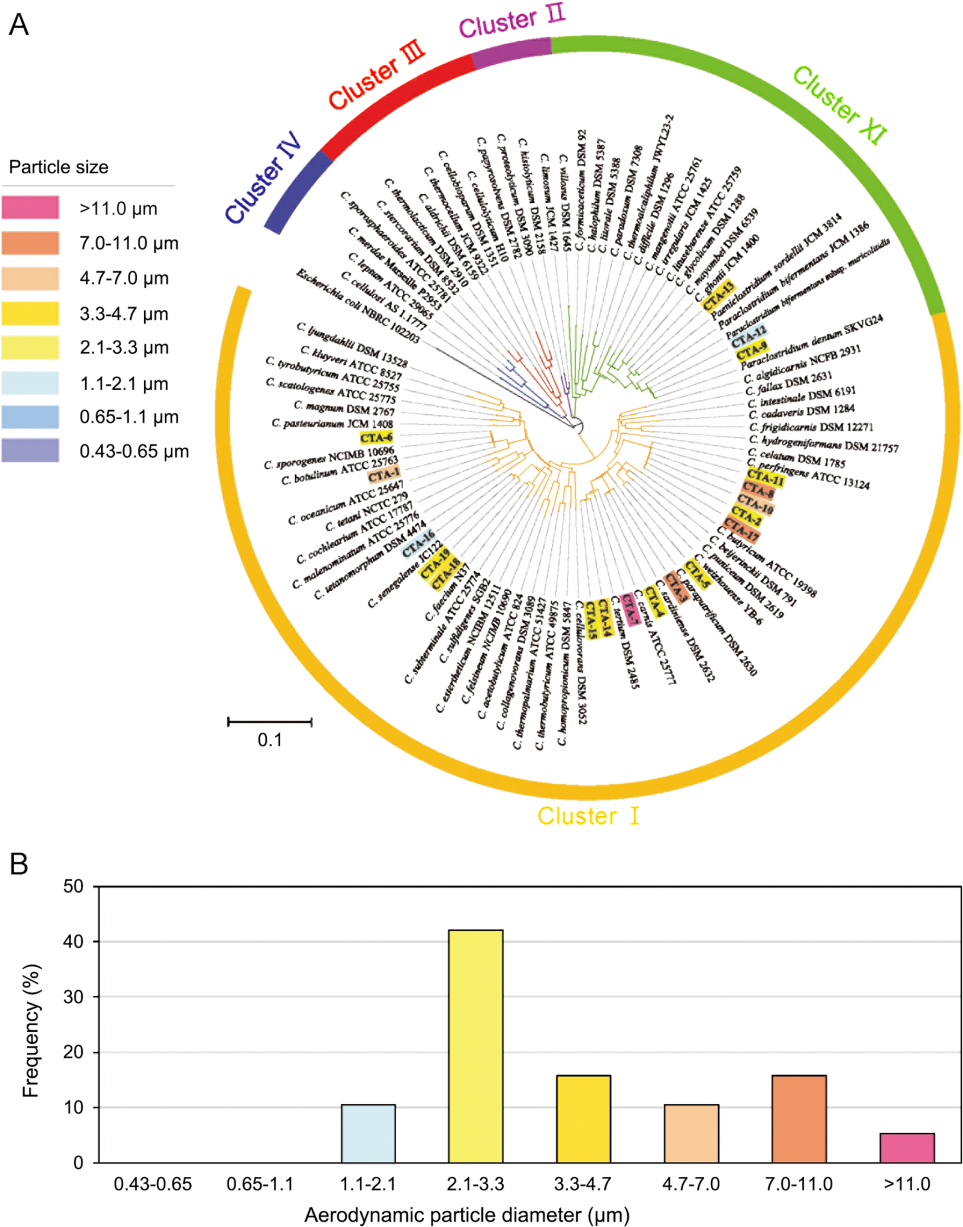
The 16S rRNA gene-based phylogenetic tree of *Clostridium* isolates from air samples collected using size-fractionated sampling. (A) Phylogenetic tree. The *Escherichia coli* NBRC 102203 sequence (AB681728) was used as the outgroup. (B) Frequencies of *Clostridium* isolates in eight size fractions.

Nineteen *Clostridium* isolates were obtained from 312 size-fractionated samples collected in each stage of the eight-stage Andersen cascade impactor during 39 air sampling campaigns over a 2-year period (Fig. 1A and Table S1), with a density of 2.5×10^–2^ cells m^–3^. Seventeen of the 19 isolates (89.5%) were found in coarse particle fractions (d_a_ >2.1‍ ‍μm) (Fig. 1B). *Clostridium* isolates dominated in the size fraction (d_a_ 2.1–3.3‍ ‍μm), accounting for eight of the isolates (42.1%). In fine particle fractions (d_a_ <2.1‍ ‍μm), two isolates (10.5%) were found in the size fraction (d_a_ 1.1–2.1‍ ‍μm). No isolates were detected in smaller particle fractions (d_a_ <1.1‍ ‍μm). Seasonal variations were not observed in the frequencies of *Clostridium* isolation.

The preliminary identification of the 19 *Clostridium* isolates was performed using PCR-based 16S rRNA gene sequencing. Isolates were confirmed as *Clostridium* spp. by a phylogenetic ana­lysis based on 16S rRNA gene sequences (Fig. 1A and Fig. S1). Sixteen isolates were in *Clostridium* cluster I (*Clostridium sensu stricto*) (Lawson and Rainey, 2016), whereas isolates CTA-9, CTA-12, and CTA-13 were in *Clostridium* cluster XI (Poehlein *et al.*, 2017).

### Genome sequencing and taxonomic characterization

**Fig. 2.**
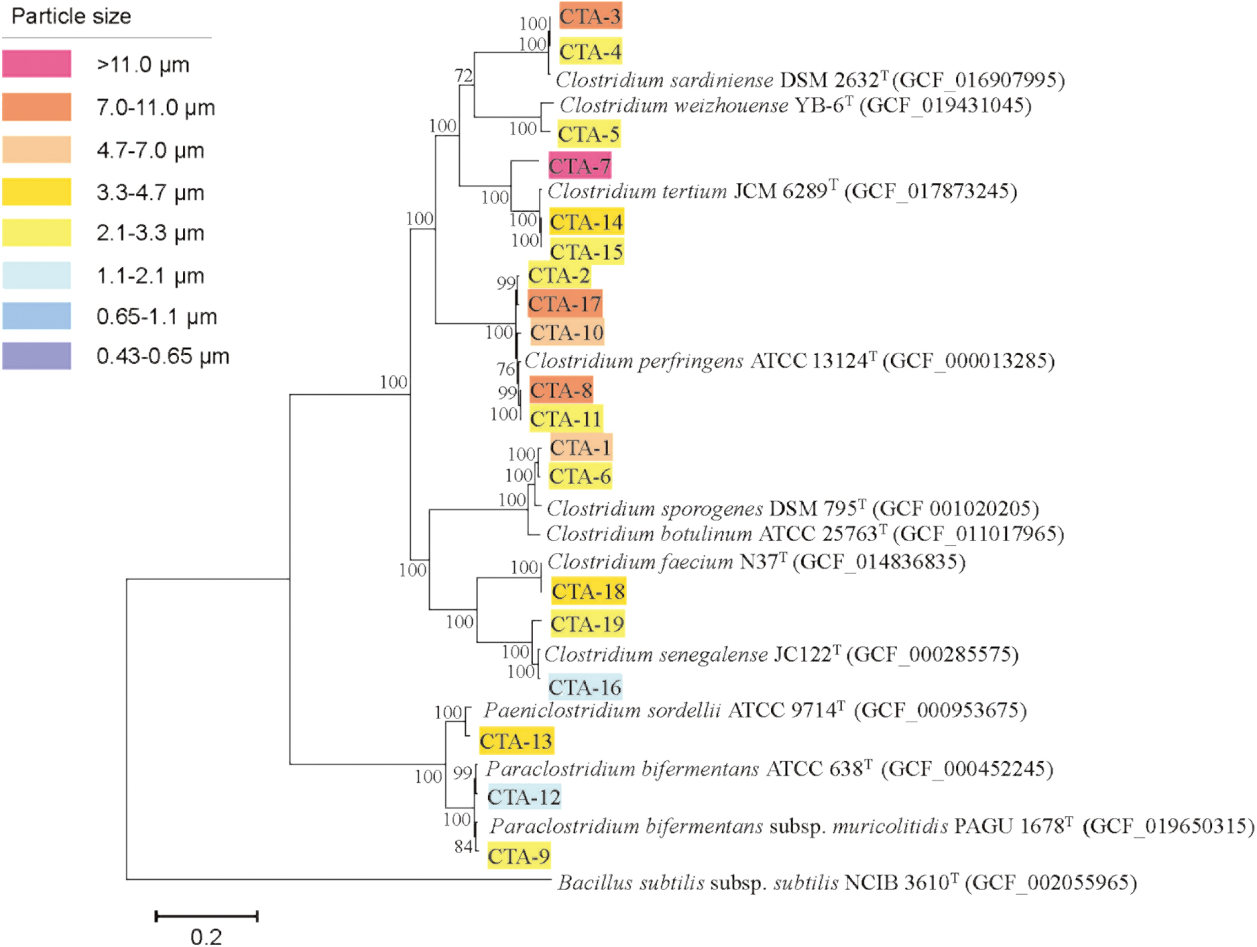
Maximum-likelihood phylogenetic tree inferred from concatenated alignments of 32 marker genes in 19 *Clostridium* genomes. The *Bacillus subtilis* subsp. *subtilis* NCIB 3610^T^ sequence was used as the outgroup.

To confirm the phylogenetic positions of the 19 airborne *Clostridium* isolates, we assembled draft genome sequences (Table S2). Genome sizes were 3.1–4.4‍ ‍Mbp, DNA G+C contents were 26.7–28.6‍ ‍mol%, and coding sequences (CDSs) were 2,750–4,186. A phylogenomic tree was constructed based on the whole-genome concatenated alignment of 32 marker genes (Fig. 2 and Table S3). The resultant tree (Fig. 2) was similar to the tree constructed based on 16S rRNA gene sequences (Fig. S1).

The classification of 19 *Clostridium* isolates with the TYGS tool identified four *C. perfringens* (CTA-2, CTA-8, CTA-10, and CTA-11), two *Clostridium sardiniense* (CTA-3 and CTA-4), two *Clostridium tertium* (CTA-14 and CTA-15), two *Paraclostridium bifermentans* (formerly known as *Clostridium bifermentans*) (CTA-9 and CTA-12), one *Paeniclostridium sordellii* (formerly known as *Clostridium sordellii*) (CTA-13), one *Clostridium senegalense* (CTA-16), and one *Clostridium faecium* (CTA-18) (Table S4). The remaining six isolates (CTA-1, CTA-5, CTA-6, CTA-7, CTA-17, and CTA-19) were potentially new species.

To increase the accuracy of identification at the species level, we calculated the ANI and dDDH values of the 19 *Clostridium* isolates with the type strains of the species of the genus *Clostridium* (Table S5). An ANI value of 95–96% and dDDH value of 70% were used to define species boundaries (Liu *et al.*, 2020). Thirteen of the 19 isolates had ANI values >95% and dDDH values >70% (CTA-2, CTA-3, CTA-4, CTA-8, CTA-9, CTA-10, CTA-11, CTA-12, CTA-13, CTA-14, CTA-15, CTA-16, and CTA-18) and belonged to the same species with which they were compared. Six isolates had dDDH values <70% (CTA-1, CTA-5, CTA-6, CTA-7, CTA-17, and CTA-19) and remained unidentified, implying that they are potentially new species.

### Detection of virulence genes

Among the 19 *Clostridium* isolates, 13 virulence genes were detected (Table 1). All isolates, except for CTA-19, harbored at least one or more virulence genes. The possession of virulence genes varied among species. For example, *C. perfringens* isolates (CTA-2, CTA-8, CTA-10, and CTA-11) had diverse repertories of virulence genes that encode alpha-toxin (*plc*), beta2-toxin (*cpb2*), theta-toxin/perfringolysin O (*pfoA*), alpha-clostripain/cysteine protease‍ ‍(*cloSI*), kappa-toxin/collagenase (*colA*), mu-toxin/hyaluronidase (*nagH*, *nagI*, *nagJ*, *nagK*, and *nagL*), and sialidase (*nanH*, *nanI*, and *nanJ*). *C. sardiniense* isolates (CTA-3 and CTA-4) possessed the *plc*, *pfoA*, *colA*, *nagK*, *nagL*, *nanH*, and *nanJ* genes. *C. tertium* isolates CTA-14 and CTA-15 possessed the *nagH*, *nagI*, and *nagK* genes.

### Detection of ARGs

ARGs were detected in 47.4% (9/19) of *Clostridium* isolates (Table 2). *C. perfringens* CTA-11 harbored multiple ARGs, *i.e.*, genes encoding tetracycline resistance (*tetA*[P], *tetB*[P]), defensin resistance (*mprF*), and erythromycin resistance (*emr*[Q]). Three *C. perfringens* isolates (CTA-2, CTA-8, and CTA-10) and *Clostridium* sp. CTA-17 possessed the defensin resistance gene *mprF*. The multi-resistance gene *cfrC* was found in three *Clostridium* sp. isolates (CTA-1, CTA-6, and CTA-7), and the macrolide resistance gene *mef*(A) was detected in *C. faecium* CTA-18.

### 16S rRNA gene amplicon sequencing

**Fig. 3.**
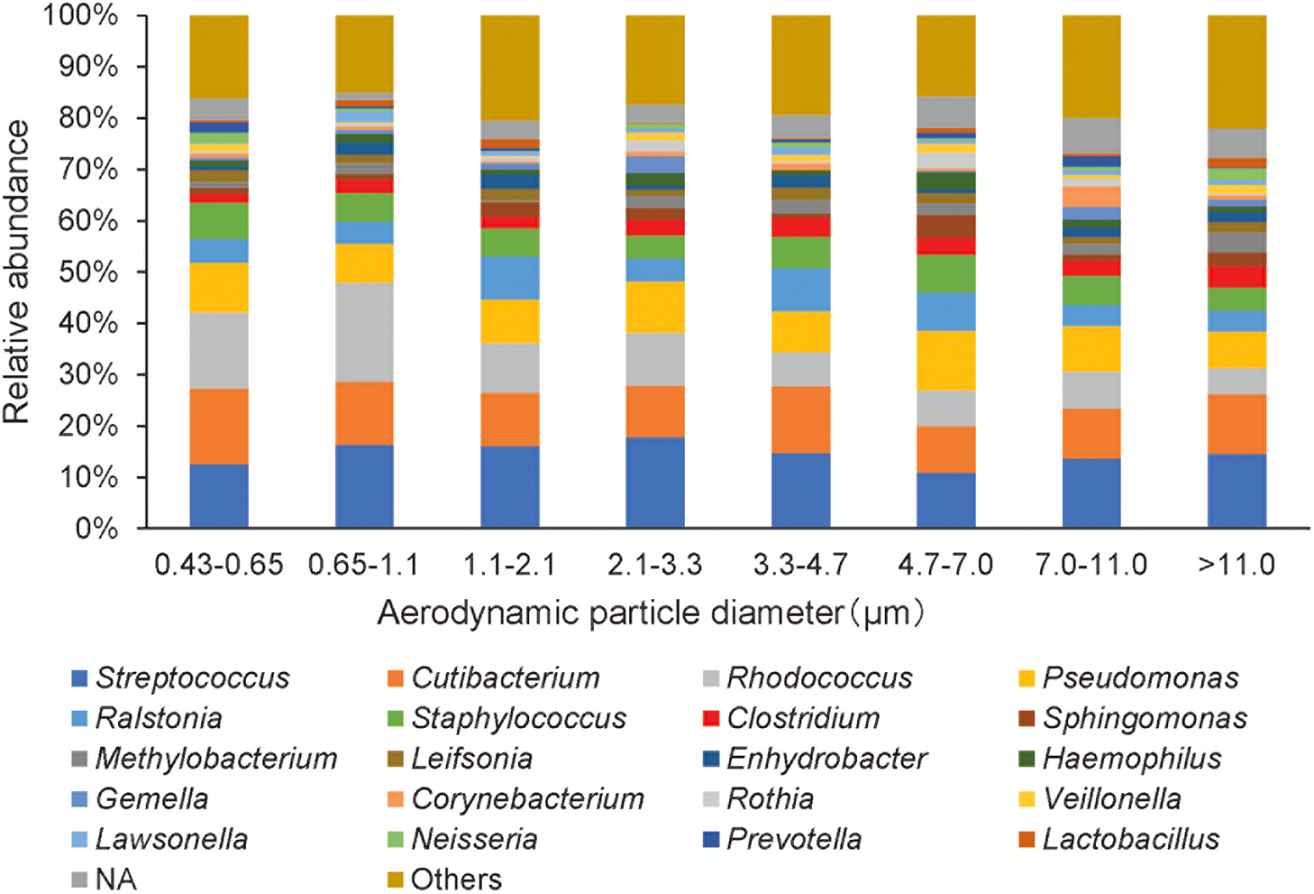
Mean relative abundance of bacterial genera in eight size fractions. NA, not assigned.

Overall, 3,698,145 raw sequence reads were obtained from 96 size-fractionated samples collected in each stage of the eight-stage Andersen cascade impactor during 12 air sampling campaigns over a 1-year period (Fig. 3, Table S1 and S6). In the present study, we primarily used culture-based approaches to study airborne *Clostridium* spp. and used culture-independent approaches for some samples and compared the results. A total of 2,359,659 clean reads (24,580 reads per sample) were clustered into 2,536 ASVs. Regarding alpha diversity, microbial richness (Chao1 index) ranged between 14 and 104, the Shannon diversity index ranged between 0.90 and 3.91, and the Simpson index ranged between 0.32 and 0.98 (Table S6). According to species annotation, 231 genera were detected. The bacterial community bar plot shows the mean relative abundance of bacterial genera from 12 replicates in each size fraction (Fig. 3 and Table S1). The dominant genera were *Streptococcus* (15.2%), *Cutibacterium* (11.6%), *Rhodococcus* (9.9%), *Pseudomonas* (8.6%), *Staphylococcus* (5.8%), *Ralstonia* (5.7%), *Clostridium* (3.1%), *Methylobacterium* (2.1%), *Sphingomonas* (2.1%), and *Leifsonia* (1.8%). The genus *Clostridium* was the seventh most abundant in mean relative abundance, being approximately 1.6-fold more abundant in coarse particles (3.6%) than in fine particles (2.3%), which was a significant difference (*P*<0.05). Seasonal variations were not observed in microbial communities.

## Discussion

The totality of spore-forming bacteria has recently been termed the “sporobiota”, emphasizing its importance and special position within the bacterial microbiota (Tetz and Tetz, 2017; Egan *et al.*, 2021; Manetsberger *et al.*, 2023; Xu *et al.*, 2023). Endospores are produced by members of the phylum *Firmicutes*, which includes the genera *Bacillus*, *Clostridium*, *Paenibacillus*, *Alicyclobacillus*, *Geobacillus*, and *Turicibacter*. The spread of the environmental sporobiota to soil, water, and air may pose significant health risks to humans and animals (Tetz and Tetz, 2017; Egan *et al.*, 2021; Manetsberger *et al.*, 2023; Xu *et al.*, 2023). These risks may increase as a result of changes in the sporobiota life cycle caused by climate change and environmental pollution. Bacterial spores contain a complete copy of the genome and act as a pool of virulence factor genes and ARGs that may transfer horizontally to other bacterial species (Poirel *et al.*, 2003; Wright, 2007; Xu *et al.*, 2023).

In the present study, we successfully cultured airborne *Clostridium* spp. The particle size distribution of the culturable airborne *Clostridium* spp. was the highest (42.1%) in the particle size range of 2.1–3.3‍ ‍μm, which corresponds to the size of particles that penetrate the secondary bronchi of the human lung. The spore size of *Clostridium* spp. was previously reported to be 1–3.5‍ ‍μm (Novak *et al.*, 2003; Orsburn *et al.*, 2008; Brunt *et al.*, 2015; Rabi *et al.*, 2017; Ertürkmen and Öner, 2023). Therefore, these findings indicate that culturable *Clostridium* spp. are often suspended as single spores in the atmosphere. The core of an endospore is sheathed from the outside by the exosporium, spore coat, outer membrane, cortex, germ cell wall, and inner membrane (Leggett *et al.*, 2012; Egan *et al.*, 2021; Xu *et al.*, 2023). Culturable *Clostridium* spp. were also found on coarse particles with diameters ≥3.3‍ ‍μm, which may have been due to cells being attached to other particles. Furthermore, *Clostridium* spp. with high oxygen tolerance may have been suspended in the atmosphere in the form of vegetative cells. For example, *C. tertium* is known to be tolerant of oxygen, while *C. perfringens* exhibits high resistance to oxygen since vegetative cells survived air exposure for 1‍ ‍h (Lew *et al.*, 1990; Boyanova *et al.*, 2024). Vegetative cells occur singly, in pairs, in short chains, or as irregular masses.

The bacterial community structure of air samples was similar to that reported in previous studies (Tanaka *et al.*, 2015, 2019, 2020; Seki *et al.*, 2024). The abundance of *Clostridium* spp. was markedly higher than that of potentially pathogenic *Legionella* spp. (approximately 0.5%) detected in a previous study (Tanaka *et al.*, 2020) using a similar ana­lysis. Furthermore, *Clostridium* spp. were detected more frequently in coarse particles (>2.1‍ ‍μm) than in fine particles (<2.1‍ ‍μm), which is consistent with previous findings (Leski *et al.*, 2011; Krishnamurthi and Chakrabarti, 2013; Liu *et al.*, 2018; Ruiz-Gil *et al.*, 2020). The peak in the detection rate for particles of 2.1–3.3‍ ‍μm obtained with the culture-based method was less pronounced than that from 16S rRNA gene amplicon sequencing. Molecular-based approaches detect DNA from dead as well as live bacteria, which may lead to different results to those derived from culture-based approaches. In the present study, dead cells may have been detected from fine particles as fragmented cells. Additionally, a PCR bias may have affected the results obtained.

Among the *Clostridium* spp. detected, *C. perfringens* was the most abundant, followed by *C. sardiniense*, *C. tertium*, *P. bifermentans*, *C. senegalense*, *C. faecium*, and *P. sordellii*. *C. perfringens* is the causative agent of many enterotoxigenic diseases in humans and animals. It is present in diverse environments, including soil, feces, fresh water, and seawater in Japan and other countries (Saito, 1990). *C. perfringens* is also an indicator of fecal contamination in the environment (Skanavis and Yanko, 2001; Abia *et al.*, 2015) and needs to be the focus of future air monitoring. Regarding the potential health risks of airborne *Clostridium*, their size distribution has raised concerns regarding lung and gastrointestinal illnesses (Mutlu *et al.*, 2018; Van Pee *et al.*, 2023). Non-traumatic *C. perfringens* gas gangrene has been reported and differs from gas gangrene associated with typical trauma or intra-abdominal penetrating wounds (Hifumi, 2020; Sasaki *et al.*, 2000; Yildiz *et al.*, 2006). These cases are often associated with underlying diseases, and lesions are generally found in the liver, gastrointestinal tract, and muscles. *C. perfringens* also rarely causes pneumonia in the absence of trauma (Patel and Mahler, 1990). Furthermore, *C. perfringens* has frequently been associated with various intestinal diseases, particularly in neonatal humans and animals (Kiu *et al.*, 2023). We suggest that airborne *C. perfringens* plays a role in the etiology of these diseases. *C. tertium*, *P. bifermentans*, and *P. sordellii*, which were detected in the present study, are also known pathogens (Moore and Lacey, 2019).

We analyzed the virulence gene profiles of the 19 *Clostridium* isolates to evaluate the distribution of virulence genes in culturable airborne *Clostridium* species. Although many toxin genes were detected in *C. perfringens* isolates, more than 20 toxin genes have been identified in this species (Kiu and Hall, 2018; Feng *et al.*, 2020). The four isolates of *C. perfringens* from this study were classified as type A because they carried only the alpha-toxin gene *plc* out of the six toxin typing genes responsible for the toxin type (Ueda *et al.*, 2022). Alpha-toxin is a membrane-damaging toxin that exhibits phospholipase C and sphingomyelinase activities and is the causative toxin of gas gangrene, which is a fast-moving and life-threatening infection involving severe pain, swelling, edema, myonecrosis, and gas production. *C. perfringens*, *C. sardiniense*, *P. bifermentans*, and *P. sordellii* also carry the *plc* gene (Wang *et al.*, 2005; Popoff and Bouvet, 2013), and similar results were obtained in this study. The theta-toxin/perfringolysin O gene *pfoA* was found in many of our isolates. Perfringolysin O is a cholesterol-binding cytolysin that forms pores through the cell membrane (Petit *et al.*, 1999; Gohari *et al.*, 2021). Together with alpha-toxin, perfringolysin O is also involved in gangrene (Popoff and Bouvet, 2013). A study on *C. perfringens*-associated necrotizing enterocolitis in preterm infants highlighted the importance of *pfoA*^+^ *C. perfringens* strains as enteric pathogens (Kiu *et al.*, 2023). The beta2-toxin gene *cpb2*, which was identified in isolate CTA-10, encodes another pore-forming toxin and has been detected in animals and humans with enteric disease (Gohari *et al.*, 2021). Other virulence genes that encode extracellular enzymes were also found, namely, the alpha-clostripain/cysteine protease gene, kappa-toxin/collagenase gene, mu-toxin genes, and sialidase genes. The contribution of these factors to pathogenicity has been the focus of intensive research (Gohari *et al.*, 2021). For example, alpha-clostripain and collagenase are not major factors contributing to virulence in clostridial myonecrosis; however, they may exert effects in *C. perfringens*-mediated disease (Kim *et al.*, 2017). Mu-toxin is considered to play a role in the pathogenesis of *C. perfringens* infections by degrading mucins and connective tissue (Goossens *et al.*, 2017). Sialidases liberate free sialic acids from various sialoglycoconjugates present on the surface of host cells or in mucus and aid intestinal colonization (Gohari *et al.*, 2021).

Airborne antimicrobial resistance, including antimicrobial-resistant bacteria and ARGs in bioaerosols, has received considerable attention worldwide (Ginn *et al.*, 2022; Gwenzi *et al.*, 2022; Xu *et al.*, 2023). Our genomic ana­lysis of culturable airborne *Clostridium* showed that *C. perfringens* isolates harbored anti-defensins genes, tetracycline resistance genes, and erythromycin resistance genes. Isolate CTA-11 possessed diverse ARGs. *mprF* is the most abundant ARG (almost 100%) in *C. perfringens* (Kiu *et al.*, 2017; Feng *et al.*, 2020; AlJindan *et al.*, 2023). Multiple peptide resistance factor (MprF) confers protection against cationic antimicrobial peptides (Assoni *et al.*, 2020; AlJindan *et al.*, 2023; Rashid *et al.*, 2023). Furthermore, the tetracycline resistance genes *tetA*(P) and *tetB*(P) and erythromycin resistance gene *erm*(Q) have been detected in *C. perfringens* (Kiu and Hall, 2018; Feng *et al.*, 2020; Yadav *et al.*, 2022). The macrolide resistance gene *mef*(A) (Soge *et al.*, 2009; Adams *et al.*, 2018) and the 23S rRNA methyltransferase gene *cfrC* (Zhang *et al.*, 2021; Karnachuk *et al.*, 2023) were identified in several *Clostridium* spp. The CfrC protein confers resistance to linezolid and phenicol antibiotics. A PCR-based ARG study on 160 *C. perfringens* isolates from water, soil, and sewage revealed *tetA*(P) (53%), *tetB*(P) (22%), *tet*(M) (8%), *erm*(B) (26%), *erm*(Q) (1%), and *mef*(A) (18%) (Soge *et al.*, 2009). A recent WGS study confirmed the presence of *mprF* (98%), *tetA*(P) (65%), and *tetB*(P) (35%) in 173 *C. perfringens* genomes from animal, human, and environmental sources (Feng *et al.*, 2020). Our results are consistent with these findings and suggest that airborne *C. perfringens* harbors similar ARGs to those found in *C. perfringens* in other environments. The occurrence of virulence and resistance genes in the environmental sporobiota arises from microevolutionary processes, such as homologous recombination, intrinsic mechanisms, and horizontal gene transfer (Xu *et al.*, 2023). Additionally, spores are crucial for maintaining and disseminating genetic information within the sporobiota.

## Conclusion

In the present study, we obtained 19 culturable *Clostridium* isolates from air samples collected by size-fractionated sampling. The size distribution of culturable* Clostridium* spp. peaked at 2.1–3.3‍ ‍μm, aligning with the spore size of *Clostridium* spp. WGS identified seven *Clostridium* species, with *C. perfringens* being the most frequent. *C. perfringens* isolates harbored numerous virulence genes and ARGs, raising concerns about their potential dissemination in the environment via atmospheric transport and horizontal gene transfer. Additionally, several potentially new species were discovered, warranting further characterization. These results highlight the potential health risks posed by the anaerobic sporobiota in the atmosphere and provide valuable insights for the prevention and control of airborne *Clostridium*. However, further studies are needed to evaluate the spatiotemporal dynamics and impact of the anaerobic sporobiota in the atmosphere.

## Citation

Seki, M., Iwamoto, R., Hou, J., Fujiyoshi, S., Maruyama, F., Furusawa, Y., et al. (2025) Size Distribution and Pathogenic Potential of Culturable Airborne *Clostridium* spp. in a Suburb of Toyama City, Japan. *Microbes Environ ***40**: ME24078.

https://doi.org/10.1264/jsme2.ME24078

## Supplementary Material

Supplementary Material

## Figures and Tables

**Table 1.** Distribution of virulence genes in 19 *Clostridium* isolates from air samples

Species	Isolate	Virulence genes												
		*plc*	*cpb2*	*pfoA*	*cloSI*	*colA*	*nagH*	*nagI*	*nagJ*	*nagK*	*nagL*	*nanH*	*nanI*	*nanJ*
*Clostridium perfringens*	CTA-2	+	–	+	+	+	+	+	+	+	+	+	+	+
	CTA-8	+	–	–	+	+	–	+	–	+	–	+	–	–
	CTA-10	+	+	+	+	+	+	+	+	+	+	+	+	+
	CTA-11	+	–	+	+	+	+	+	–	+	–	+	–	–
*Clostridium sardiniense*	CTA-3	+	–	+	–	+	–	–	–	+	+	+	–	+
	CTA-4	+	–	+	–	+	–	–	–	+	+	+	–	+
*Clostridium tertium*	CTA-14	–	–	–	–	–	+	+	–	+	–	–	–	–
	CTA-15	–	–	–	–	–	+	+	–	+	–	–	–	–
*Clostridium senegalense*	CTA-16	–	–	–	+	–	–	–	–	–	–	–	–	–
*Clostridium faecium*	CTA-18	–	–	+	–	–	–	–	–	–	–	–	–	–
*Paraclostridium bifermentans*	CTA-12	+	–	+	–	–	–	–	–	–	–	–	–	–
*Paraclostridium bifermentans* subsp.* muricolitidis*	CTA-9	+	–	+	–	+	–	–	–	–	–	–	–	–
*Paeniclostridium sordellii*	CTA-13	+	–	+	–	–	–	–	–	–	–	+	–	–
Unidentified *Clostridium* species	CTA-1	–	–	–	+	+	–	–	–	–	–	–	–	–
	CTA-5	+	–	+	–	–	–	–	–	–	–	–	–	–
	CTA-6	–	–	–	+	+	–	–	–	–	–	–	–	–
	CTA-7	–	–	+	–	–	–	+	–	–	–	–	–	–
	CTA-17	+	–	+	+	+	+	+	+	–	+	+	+	+
	CTA-19	–	–	–	–	–	–	–	–	–	–	–	–	–

**Table 2.** Distribution of antibiotic resistance genes in 19 *Clostridium* isolates from air samples

Species	Isolate	Antibiotic resistance genes					
		*tetA*(P)	*tetB*(P)	*mef*(A)	*cfrC*	*mprF*	*erm*(Q)
*Clostridium perfringens*	CTA-2	–	–	–	–	+	–
	CTA-8	–	–	–	–	+	–
	CTA-10	–	–	–	–	+	–
	CTA-11	+	+	–	–	+	+
*Clostridium sardiniense*	CTA-3	–	–	–	–	–	–
	CTA-4	–	–	–	–	–	–
*Clostridium tertium*	CTA-14	–	–	–	–	–	–
	CTA-15	–	–	–	–	–	–
*Clostridium senegalense*	CTA-16	–	–	–	–	–	–
*Clostridium faecium*	CTA-18	–	–	+	–	–	–
*Paraclostridium bifermentans*	CTA-12	–	–	–	–	–	–
*Paraclostridium bifermentans* subsp. *muricolitidis*	CTA-9	–	–	–	–	–	–
*Paeniclostridium sordellii*	CTA-13	–	–	–	–	–	–
Unidentified *Clostridium* species	CTA-1	–	–	–	+	–	–
	CTA-5	–	–	–	–	–	–
	CTA-6	–	–	–	+	–	–
	CTA-7	–	–	–	+	–	–
	CTA-17	–	–	–	–	+	–
	CTA-19	–	–	–	–	–	–
