# Supplementary material for: Size Distribution and Pathogenic Potential of Culturable Airborne Clostridium spp. in a Suburb of Toyama City, Japan
Source: Microbes Environ. 2025 Feb 6;40(1):ME24078. doi: 10.1264/jsme2.ME24078 (PMC11946412; doi:10.1264/jsme2.ME24078)
Supplement: Supplementary file 1 — Supplementary Material [file 40_24078_s1.pdf]

Supplementary material

Size Distribution and Pathogenic Potential of Culturable Airborne *Clostridium* spp. in a Suburb of Toyama City, Japan

Makoto Seki[1], Reika Iwamoto[1], Jianjian Hou[2], So Fujiyoshi[2], Fumito Maruyama[2], Yukihiro Furusawa[3], Shigehiro Kagaya[1], Akihiro Sakatoku[1], Shogo Nakamura[1], Daisuke Tanaka[1]*

[1]Graduate School of Science and Engineering, University of Toyama, 3190 Gofuku, Toyama, Toyama 930-8555, Japan

[2]Center for the Planetary Health and Innovation Science (PHIS), The IDEC Institute, Hiroshima University, 1-3-2 Kagamiyama, Higashi-Hiroshima, Hiroshima 739-8511, Japan

[3]Department of Pharmaceutical Engineering, Faculty of Engineering, Toyama Prefectural University, 5180 Kurokawa, Imizu, Toyama 939-0398, Japan

*Corresponding author. E-mail: tanakada@sci.u-toyama.ac.jp; Tel: +81-76-445-6673

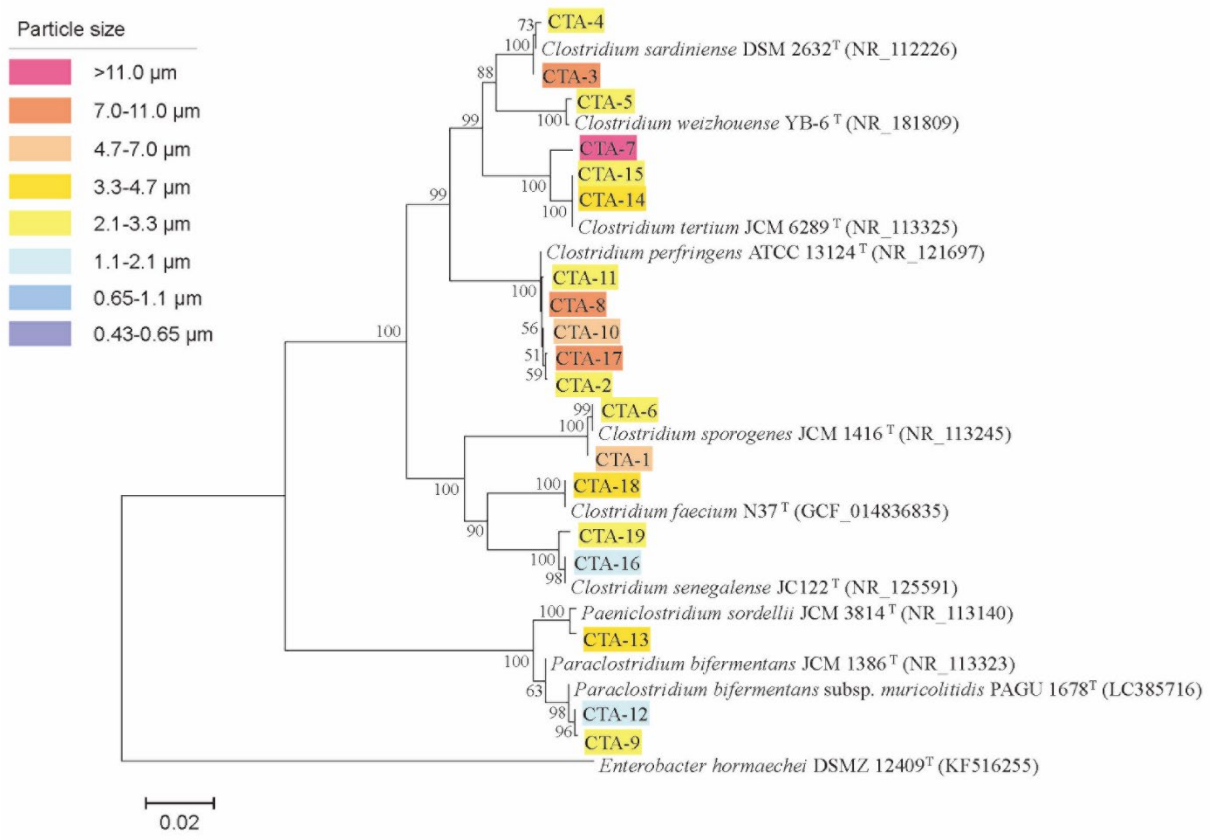

Fig. S1. Neighbor-joining phylogenetic tree based on 16S rRNA gene sequences, showing the relationships between airborne *Clostridium* isolates and closely related species. Bootstrap values >50% are indicated for each node (1000 replicates). Scale bar indicates 0.02 substitutions per nucleotide position.

Table S1. Sample collection information

Sampling date	Isolate	Sample for 16S rRNA gene amplicon sequencing	Average temperature (℃)	Average humidity (%)	Average wind speed (m s^{-1})	Predominant wind direction	Precipitation (mm)	Snow depth (cm)
2021/4/22-23			12.8	64.5	2.5	NNE	0.0	0.0
2021/5/20-21	CTA-1, CTA-2		17.6	94.7	2.4	NNE	1.8	0.0
2021/6/22-23			22.0	75.8	2.8	W	0.0	0.0
2021/8/26-27	CTA-3, CTA-4		28.6	73.2	3.4	W	0.0	0.0
2021/9/2-3	CTA-5		22.6	82.3	2.4	NNE	0.0	0.0
2021/9/13-14	CTA-6		21.1	75.7	2.5	NNE	0.0	0.0
2021/10/10-11			26.0	54.7	5.2	S	0.0	0.0
2021/11/1-2	CTA-7, CTA-8, CTA-9		15.7	79.2	2.1	NW	0.0	0.0
2021/11/16-17			10.8	73.3	2.6	NW	0.0	0.0
2021/11/29-30	CTA-10, CTA-11		8.7	56.7	3.4	NW	0.0	0.0
2021/12/6-7	CTA-12	1207-0–1207-7	8.4	97.5	1.4	SSW	0.3	0.0
2022/1/10-11		0110-0–0110-7	4.7	78.8	2.2	SW	0.5	1.0
2022/1/17-18			0.8	94.2	2.7	SSW	1.0	23.9
2022/1/31-2/1		0131-0–0131-7	2.6	74.9	2.1	SW	0.0	12.2
2022/2/7-8			1.5	75.8	2.2	SSW	0.0	26.3
2022/3/7-8		0307-0–0307-7	3.6	71.9	2.2	SW	0.0	0.0
2022/3/14-15			11.8	81.6	3.9	WSW	0.8	0.0
2022/3/28-29	CTA-13	0328-0–0328-7	8.1	55.8	2.6	NE	0.0	0.0
2022/4/4-5			10.9	79.8	2.1	N	0.0	0.0
2022/4/18-19			13.0	69.9	2.1	SSW	0.0	0.0
2022/5/9-10		0509-0–0509-7	13.3	55.6	3.0	NNE	0.0	0.0
2022/5/23-24			18.2	71.3	2.4	NNE	0.0	0.0
2022/6/6-7			18.2	87.3	2.9	WSW	0.8	0.0
2022/6/13-14	CTA-14, CTA-15	0613-0–0613-7	18.5	77.7	3.0	NNE	0.0	0.0
2022/7/11-12		0711-0–0711-7	27.6	81.7	2.1	NNE	0.4	0.0
2022/7/25-26	CTA-16		28.3	76.0	1.8	NNE	0.0	0.0
2022/8/22-23		0822-0–0822-7	29.1	75.0	1.8	N	0.0	0.0
2022/8/29-30			24.8	76.0	1.8	N	0.0	0.0
2022/9/5-6		0905-0–0905-7	30.2	59.6	7.6	SSW	0.0	0.0
2022/9/12-13			27.5	82.0	1.9	NNE	0.0	0.0
2022/10/3-4		1003-0–1003-7	25.8	50.7	5.8	S	0.0	0.0
2022/10/17-18			16.7	89.8	2.6	SW	0.4	0.0
2022/11/7-8	CTA-17	1107-0–1107-7	13.0	67.4	2.6	SSW	0.4	0.0
2022/11/14-15			11.9	77.5	2.1	SSW	0.0	0.0
2022/12/12-13			8.8	70.0	3.2	S	0.1	0.0
2023/1/16-17	CTA-18		2.1	90.0	2.7	SSW	0.2	0.0
2023/2/20-21			1.9	82.2	3.1	W	0.2	0.0
2023/3/20-21			13.1	47.2	3.4	S	0.0	0.0
2023/3/23-24	CTA-19		14.5	92.6	2.7	W	0.8	0.0

Table S2. Characteristics of the genomes of 19 *Clostridium* isolates included in this study

Isolate	Draft Genome size (bp)	G + C (mol%)	No. of CDSs	No. of contigs	Contig N50	Coding Ratio (%)
CTA-1	4,076,765	27.8	3,728	75	187,204	82.4
CTA-2	3,674,782	27.9	3,258	81	203,793	81.1
CTA-3	3,734,314	28.6	3,572	36	383,344	86.1
CTA-4	3,734,422	28.6	3,573	34	533,409	86.1
CTA-5	4,254,465	26.7	3,753	40	376,550	81.4
CTA-6	4,362,825	27.9	4,186	40	311,297	82.7
CTA-7	3,930,872	28.1	3,644	38	479,570	85.7
CTA-8	3,101,422	28.1	2,769	100	84,024	83.0
CTA-9	3,623,396	28.1	3,494	52	297,909	86.6
CTA-10	3,471,532	28.0	3,068	121	166,673	82.8
CTA-11	3,098,515	28.1	2,750	140	56,993	82.4
CTA-12	4,070,206	28.4	4,093	65	306,581	86.9
CTA-13	3,627,123	27.2	3,562	55	307,801	86.3
CTA-14	4,057,166	28.2	3,673	261	204,843	82.5
CTA-15	3,964,695	27.8	3,659	61	210,294	84.3
CTA-16	3,836,774	26.9	3,463	73	225,121	81.0
CTA-17	3,328,837	28.0	2,949	36	342,744	84.1
CTA-18	3,737,000	28.3	3,414	83	125,863	80.8
CTA-19	3,939,733	27.1	3,477	112	110,249	79.8

Table S3. Thirty-two marker genes used to construct the phylogenetic tree based on whole-genome sequences

TIGR number	Gene	Function	Description
TIGR00012	*L29*	Protein synthesis	ribosomal protein uL29
TIGR00019	*prfA*	Protein synthesis	peptide chain release factor 1
TIGR00048	*rRNA_mod_RlmN*	Protein synthesis	23S rRNA (adenine(2503)-C(2))-methyltransferase
TIGR00060	*L18_bact*	Protein synthesis	ribosomal protein uL18
TIGR00184	*purA*	Purines, pyrimidines, nucleosides, and nucleotides	adenylosuccinate synthase
TIGR00244	*TIGR00244*	Regulatory functions	transcriptional regulator NrdR
TIGR00436	*era*	Protein synthesis	GTP-binding protein Era
TIGR00447	*pth*	Protein synthesis	aminoacyl-tRNA hydrolase
TIGR00459	*aspS_bact*	Protein synthesis	aspartate--tRNA ligase
TIGR00468	*pheS*	Protein synthesis	phenylalanine--tRNA ligase, alpha subunit
TIGR00521	*coaBC_dfp*	Biosynthesis of cofactors, prosthetic groups, and carriers	phosphopantothenoylcysteine decarboxylase / phosphopantothenate--cysteine ligase
TIGR00615	*recR*	DNA metabolism	recombination protein RecR
TIGR00635	*ruvB*	DNA metabolism	Holliday junction DNA helicase RuvB
TIGR00855	*L12*	Protein synthesis	ribosomal protein bL12
TIGR01011	*rpsB_bact*	Protein synthesis	ribosomal protein uS2
TIGR01021	*rpsE_bact*	Protein synthesis	ribosomal protein uS5
TIGR01044	*rplV_bact*	Protein synthesis	ribosomal protein uL22
TIGR01049	*rpsJ_bact*	Protein synthesis	ribosomal protein uS10
TIGR01059	*gyrB*	DNA metabolism	DNA gyrase, B subunit
TIGR01083	*nth*	DNA metabolism	endonuclease III
TIGR01163	*rpe*	Energy metabolism	ribulose-phosphate 3-epimerase
TIGR01169	*rplA_bact*	Protein synthesis	ribosomal protein uL1
TIGR01171	*rplB_bact*	Protein synthesis	ribosomal protein uL2
TIGR01296	*asd_B*	Amino acid biosynthesis	aspartate-semialdehyde dehydrogenase
TIGR02027	*rpoA*	Transcription	DNA-directed RNA polymerase, alpha subunit
TIGR02386	*rpoC_TIGR*	Transcription	DNA-directed RNA polymerase, beta' subunit
TIGR03263	*guanyl_kin*	Purines, pyrimidines, nucleosides, and nucleotides	guanylate kinase
TIGR03594	*GTPase_EngA*	Protein synthesis	ribosome associated GTPase EngA
TIGR03631	*uS13_bact*	Protein synthesis	ribosomal protein uS13
TIGR03632	*uS11_bact*	Protein synthesis	ribosomal protein uS11
TIGR03635	*uS17_bact*	Protein synthesis	ribosomal protein uS17
TIGR03723	*T6A_TsaD_YgjD*	Protein synthesis	tRNA threonylcarbamoyl adenosine modification protein TsaD

Table S4. Type Strain Genome Server (TYGS) analysis

Isolate	Result
CTA-1	potential new species
CTA-2	*Clostridium perfringens*
CTA-3	*Clostridium sardiniense*
CTA-4	*Clostridium sardiniense*
CTA-5	potential new species
CTA-6	potential new species
CTA-7	potential new species
CTA-8	*Clostridium perfringens*
CTA-9	*Paraclostridium bifermentans*
CTA-10	*Clostridium perfringens*
CTA-11	*Clostridium perfringens*
CTA-12	*Paraclostridium bifermentans*
CTA-13	*Paeniclostridium sordellii*
CTA-14	*Clostridium tertium*
CTA-15	*Clostridium tertium*
CTA-16	*Clostridium senegalense*
CTA-17	potential new species
CTA-18	*Clostridium faecium*
CTA-19	potential new species

Table S5. Digital DNA–DNA hybridization (dDDH) and average nucleotide identity (ANI) values between *Clostridium* isolates and their closest phylogenetic neighbors

Isolate	Type strain	dDDH (%)	ANI (%)
CTA-1	*Clostiridium sporogenes* NCIMB 10696	59.6 (56.8 - 62.4)	**95.08**
CTA-2	*Clostoridium perfringens* ATCC 13124	**70.5 (67.5 - 73.4)**	**96.50**
CTA-3	*Clostridium sardiniense* DSM 2632	**87.3 (84.7 - 89.5)**	**98.57**
CTA-4	*Clostrodium sardiniense* DSM 2632	**87.3 (84.7 - 89.5)**	**98.55**
CTA-5	*Clostridium weizhouense* YB-6	50.1 (47.4 - 52.7)	93.40
CTA-6	*Clostridium sporogenes* NCIMB 10696	60.0 (57.2 - 62.8)	**95.08**
CTA-7	*Clostridium tertium* DSM 2485	27.6 (25.2 - 30.1)	84.21
CTA-8	*Clostridium perfringens* ATCC 13124	**85.5 (82.8 - 87.8)**	**98.36**
CTA-9	*Paraclostridium bifermentans* subsp. *muricolitidis* PAGU1678	**76.6 (73.7 - 79.4)**	**97.33**
CTA-10	*Clostridium perfringens* ATCC 13124	**74.4 (71.4 - 77.2)**	**97.20**
CTA-11	*Clostridium perfringens* ATCC 13124	**85.2 (82.5 - 87.6)**	**98.31**
CTA-12	*Paraclostridium bifermentans* DSM 14991	**74.4 (71.4 - 77.2)**	**97.16**
CTA-13	*Paeniclostridium sordellii* ATCC 9714	**73.6 (70.6 - 76.4)**	**96.79**
CTA-14	*Clostridium tertium* DSM 2485	**82.6 (79.7 - 85.1)**	**98.14**
CTA-15	*Clostridium tertium* DSM 2485	**82.6 (79.8 - 85.2)**	**98.16**
CTA-16	*Clostridium senegalense* JC122	**79.0 (76.1 - 81.7)**	**97.97**
CTA-17	*Clostridium perfringens* ATCC 13124	70.0 (67.0 - 72.8)	**96.37**
CTA-18	*Clostridium faecium* N37	**97.6 (96.6 - 98.3)**	**99.84**
CTA-19	*Clostridium senegalense* JC122	60.4 (57.5 - 63.2)	**95.05**

Bold numbers indicate dDDH values >70% and/or ANI values >95%.

Table S6. Alpha diversity estimated using Chao1, Shannon, and Simpson indices at the amplicon sequence variants (ASV) level

Sample	Raw sequences	Number of clean sequences	Chao1	Shannon	Simpson
1207-0	31742	18193	33	2.95	0.94
1207-1	32450	19610	50	3.31	0.95
1207-2	39218	17224	55	3.23	0.94
1207-3	38290	25588	95	3.78	0.97
1207-4	39202	28351	54	3.31	0.95
1207-5	42639	15282	59	2.56	0.83
1207-6	30934	19275	42	3.15	0.94
1207-7	38035	25660	54	3.25	0.95
0110-0	31020	22461	34	2.96	0.93
0110-1	44354	26745	35	3.11	0.94
0110-2	44907	28119	33	2.75	0.90
0110-3	36444	25646	36	2.88	0.93
0110-4	44806	32580	33	3.04	0.94
0110-5	60190	38573	36	3.01	0.94
0110-6	47132	33052	36	2.86	0.91
0110-7	53194	32776	43	3.03	0.94
0131-0	49575	27052	41	3.14	0.95
0131-1	50826	34663	56	3.31	0.96
0131-2	45899	26554	25	2.36	0.87
0131-3	42194	27743	38	2.95	0.93
0131-4	36306	23192	34	3.28	0.96
0131-5	36831	20691	42	3.19	0.95
0131-6	34014	18219	31	3.07	0.94
0131-7	29828	20202	25	2.76	0.91
0307-0	32133	18621	45	3.12	0.94
0307-1	28819	12610	35	3.24	0.96
0307-2	47442	26339	69	3.63	0.97
0307-3	51061	17848	43	3.29	0.95
0307-4	40419	23257	48	3.35	0.95
0307-5	81977	50971	84	3.55	0.95
0307-6	40163	25572	41	3.10	0.94
0307-7	51049	31664	25	2.59	0.90

Table S6 (continued)

Sample	Raw sequences	Number of clean sequences	Chao1	Shannon	Simpson
0328-0	46244	25897	73	3.69	0.96
0328-1	50198	27879	44	2.87	0.92
0328-2	45770	28812	32	3.07	0.94
0328-3	47111	33026	33	3.15	0.95
0328-4	47500	33657	34	2.81	0.93
0328-5	44914	30760	41	3.01	0.94
0328-6	44522	31015	38	2.90	0.92
0328-7	47848	30337	104	3.38	0.91
0509-0	46338	22429	63	3.42	0.95
0509-1	42805	28989	54	3.19	0.93
0509-2	41457	26651	44	3.14	0.94
0509-3	48244	31217	46	3.06	0.92
0509-4	33707	20008	35	3.04	0.93
0509-5	40311	22238	40	2.79	0.91
0509-6	31219	13520	28	2.83	0.92
0509-7	31726	19709	47	3.25	0.94
0613-0	37742	24000	32	2.85	0.92
0613-1	35555	20566	36	3.05	0.94
0613-2	47981	30704	41	3.03	0.94
0613-3	44601	28307	54	3.28	0.95
0613-4	46689	23803	46	3.12	0.93
0613-5	44553	31896	45	3.11	0.95
0613-6	47152	34609	51	2.25	0.71
0613-7	47115	30129	51	2.04	0.66
0711-0	31776	22302	45	3.03	0.94
0711-1	38631	29559	62	3.30	0.95
0711-2	35065	27070	19	2.02	0.84
0711-3	29621	20060	74	3.33	0.95
0711-4	29229	19259	63	3.45	0.95
0711-5	34730	27488	53	3.34	0.95
0711-6	37932	30849	65	3.08	0.92
0711-7	34201	26889	49	3.07	0.93

Table S6 (continued)

Sample	Raw sequences	Number of clean sequences	Chao1	Shannon	Simpson
0822-0	41784	24612	56	3.26	0.94
0822-1	39775	30501	59	3.60	0.97
0822-2	36344	27214	65	3.57	0.97
0822-3	44426	11672	46	3.32	0.95
0822-4	31135	16959	51	3.48	0.96
0822-5	25278	14376	36	3.36	0.96
0822-6	24711	9002	33	3.19	0.95
0822-7	24403	16308	75	3.72	0.96
0905-0	36660	25039	64	3.60	0.96
0905-1	32774	21888	58	3.54	0.96
0905-2	30772	18146	49	3.46	0.96
0905-3	41284	24527	67	3.53	0.96
0905-4	37051	17484	71	3.91	0.98
0905-5	30451	20766	32	2.96	0.94
0905-6	31068	24187	42	2.81	0.91
0905-7	32983	22711	70	3.60	0.96
1003-0	32378	24442	37	2.54	0.90
1003-1	32262	25803	18	1.74	0.77
1003-2	18099	13320	27	2.74	0.91
1003-3	27593	20286	25	2.33	0.85
1003-4	23933	17300	21	1.79	0.69
1003-5	26079	19886	23	1.65	0.61
1003-6	34105	25220	17	0.90	0.32
1003-7	24530	18067	33	2.31	0.80
1107-0	30224	18040	22	2.41	0.90
1107-1	28395	18970	30	2.94	0.94
1107-2	36797	28438	20	2.45	0.90
1107-3	35068	27201	26	2.57	0.90
1107-4	32279	24914	14	1.52	0.70
1107-5	35863	28100	44	2.97	0.93
1107-6	38508	29220	18	2.20	0.87
1107-7	35553	27093	23	2.14	0.86
